# Delivery of miR-26a Using an Exosomes-Based Nanosystem Inhibited Proliferation of Hepatocellular Carcinoma

**DOI:** 10.3389/fmolb.2021.738219

**Published:** 2021-09-06

**Authors:** Shaya Mahati, Xiangjun Fu, Xuexian Ma, Hua Zhang, Lei Xiao

**Affiliations:** ^1^ Department of Oncology, The First Affiliated Hospital of Xinjiang Medical University, Urumqi, China; ^2^ Department of Otolaryngology Head and Neck Surgery, Guangdong Second Provincial General Hospital, Guangzhou, China; ^3^ Department of Infection, The First Affiliated Hospital of Xinjiang Medical University, Urumqi, China

**Keywords:** drug delivery, Glypican 3, exosomes, miR-26a, nanosystem

## Abstract

**Background:** MicroRNA (abbreviated miRNA)-based treatment holds great promise for application as clinical antitumor therapy, but good carriers for delivery of the miRNA drug are lacking. Exosomes secreted by mesenchymal stem cells (MSCs) have proved to be safe, and exogenously modified exosomes may potentially represent an excellent drug delivery vehicle.

**Methods:** In this study, we designed a delivery nano system using single-stranded variable fragment (scFv)-modified exosomes derived from human cord blood MSCs. Genetic engineering technology was used to obtain anti-Glypican 3 (GPC3) scFv-modified exosomes, which were then loaded with miR-26a mimics through electroporation.

**Results:** Results of electron microscopy and dynamic light scattering indicated that the diameter of the drug-carrying exosomes was about 160 nm. Furthermore, anti-GPC3 scFv-modified exosomes effectively delivered miR-26a to GPC3-positive hepatocellular carcinoma cells, thereby inhibiting cell proliferation and migration by regulating the expression of downstream target genes of miR-26a. The exosomes-based nano system displayed favorable anti-tumor effect *in vivo* with no obvious side effects.

**Conclusion:** Our data provided a new perspective for the use of exosome delivery systems for miRNA-based antitumor therapy.

## Introduction

MicroRNAs (miRNAs) are small (20–22 nucleotides) non-coding RNA molecules that bind to partially complementary mRNA sequences and lead to degradation or translation inhibition of target genes (Rottiers and Näär, 2012). Increasing evidence has indicated that miR-26a plays an important role as an anti-oncogene in the development of a wide range of malignant tumors. miRNA-26a is found to be significantly downregulated in human hepatocellular carcinoma cells and negatively regulate both cell proliferation and of the cell cycle (Chen et al., 2011; Dang et al., 2012). Moreover, miRNA replacement therapy has been proposed as a promising treatment strategy for the treatment of malignant tumors. However, although miRNA-based approaches may ultimately prove effective, their clinical application has been hampered by the lack of a suitable delivery system.

Exosomes are cell-secreted nanoscale vesicles (∼100 nm) that have a key role in cell-to-cell material transport and signaling (Sluijter et al., 2014; Than et al., 2018). Exosomes are similar in size and function to synthetic nanoparticles, but as natural endogenous transporters, they have the advantages of low toxicity, lack of immunogenicity, and good permeability; thus, they may represent more promising drug delivery vehicles (Sun et al., 2013). Tumor cells release exosomes, which carry miRNAs, a phenomenon that has be used clinically in tumor diagnostics, tumor cells can also absorb miRNAs secreted by donor cells and function as exosome recipient cells (Skog et al., 2008). These data suggest that exosomes are natural carriers for miRNAs and could be used as targeted RNA drug delivery systems. However, little is known about the usefulness of exosomes as vectors in cancer treatment.

Glypican 3 (GPC3) is a heparan sulfate glycoprotein on the surface of cell membranes and is a specific antigen associated with cancer (Kolluri and Ho, 2019). GPC3 is upregulated in various types of solid tumors, but its expression is very low in normal adult tissues (Zhou et al., 2018). Thus, GPC3 is considered to be a potential target for the treatment of tumors. The development of nucleic acid-based drugs has evolved considerably over the years, and the current challenge is how to retain sufficient levels in the circulation after injection to precisely target the lesion while avoiding damage to normal cells (Hanisch and Kettenmann, 2007). The single-stranded variable fragment (scFv) retains antigen-binding activity and features low molecular weight and low immunogenicity, making it an excellent drug delivery tool with targeting capabilities (Ahmad et al., 2012).

In this study, we evaluated exosomes as drug delivery nanosystem in a cancer model. Anti-GPC3 scFv-modified exosomes effectively deliver miR-26a to GPC3-expressing hepatocellular carcinoma (HCC) cells. Our findings suggest that exosomes targeting a tumor antigen could provide a platform for miRNA replacement therapies.

## Materials and Methods

### Cell Culture

HepG2 and Huh7 (HCC cells) and 293T (human embryonic kidneys cells) were provided by the American Type Culture Collection (Manassas, VA, United States) and cultured in the RPMI 1640 complete medium (Grand Island, NY, United States) containing 10% fetal bovine serum (FBS; Hyclone). Immortalized human cord blood mesenchymal stromal cells (cbMSC-hTERT) were obtained from ABM (Cat. No.: T0016; Canada) and cultured in α-MEM (Hyclone, Logan, UT, United States) with 20% FBS and a 4 ng/ml basic-fibroblast growth factor (bFGF; R&D Systems, Minneapolis, MN, United States). All cell lines were cultured in an incubator (37°C, 5% CO_2_).

### Preparation of Exosomes

Exosomes were isolated from the cellular supernatant of mesenchymal stem cell (MSC) lines cultured in serum-free Dulbecco’s Modified Eagle Medium (1% GlutaMax, Invitrogen, Carlsbad, CA, United States) for 48 h. The supernatant was centrifuged at 2,000 × g for 20 min and then at 10,000 × g for 30 min to remove cell debris. Exosomes were pelleted in small amounts by ultracentrifugation at 120,000 × g for 70 min and then washed once with phosphate buffered saline (PBS). The protein content of exosomes was measured using a rapid protein measurement kit (Wako Pure Chemicals, Osaka, Japan). The average amount of exosomes in 100 ml of the supernatant (1 × 10^7^ cells; *n* = 5) was 75.2 μg.

### Generation of Anti-Glypican 3 Single-Stranded Variable Fragment-Modified Mesenchymal Stem Cells

The coding sequence of the anti-GPC3 scFv was based on an anti-GPC3 antibody (clone GC33). A fusion gene (containing 6× His tag, anti-gpc3-scFv, full-length LAMP2B, a self-cleaved P2A linker, and puromycin) was synthesized and inserted into a pLVX-expression vector (Takara Bio, United States), which was then packaged to obtain lentivirus particles using LV-MAX Lentiviral Packaging Mix (Thermo Fisher Scientific, United States). After transduction, MSC cells expressing the scFv fusion gene (scFv-MSCs) were selected with 3 μg/ml puromycin (Invitrogen, Carlsbad, CA, United States).

### Flow Cytometry Analysis

The expression of anti-GPC3 scFV on the surface of MSC cells was confirmed by flow cytometry. 1 × 10^6^ unmodified negative control MSCs (nc-MSCs) and anti-GPC3 scFv-modified MSCs (scFv-MSCs) were collected. After being washed once with PSB, cells were incubated with 0.5 μg FITC-labeled human GPC3 protein (Fc Tag,Cat. No. GP3-HF258, Acrobiosystems) at 4°C for 30 min. To detect the expression of the GPC3 antigen in HCC cells, an APC-labeled anti-glypican 3 antibodies (clone 024, ab275695, abcam) was used. Fluorescence intensity was measured using the Cytoflex flow cytometer (Beckman Coulter, Brea, CA, United States).

### Engineering Exosomes With miR-26a Mimics

The miR-26a mimics (5’-UUC AAG UAA UCC AGG AUA GGC U-3’; Sigma-Aldrich Co. LLC) and negative control (5′-GGU UCG UAC GUA CAC UGU UCA-3′; Sigma-Aldrich Co. LLC) were loaded into MSC exosomes using the Neon electroporation system (Life Technologies, Carlsbad, CA, United States) as reported previously (Zhang et al., 2017). Briefly, after ultracentrifugation, exosomes were dissolved in the R Buffer to ensure a concentration of at least 1 mg/ml, and 10 μL miR-26a mimic (0–50 nM) was then added to 90 μL of the electro transfer solution at a mass ratio of 1:1. This mixture was electrically transformed (1,000 V, 10 ms, 2 pulses), and 1 ml of PBS was added before incubation at 37°C for 1 h to promote exosome repair. Engineered exosomes with miR-26a mimics (Exo^miR-26a^) were obtained by collecting exosomes after ultrasonication.

### The Particle Size of Stranded Variable Fragment-Exo^miR-26a^ Was Evaluated by Dynamic Light Scattering

The sample was prepared in the sample particle reserve solution as described in previous studies (Liang et al., 2018). A homogeneous solution was obtained by vertexing, and dynamic light scattering (DLS) was used to evaluate the homogeneous solution (1.5 ml) by transferring the sample to a square colorimetric cup, which was then subjected to the DynaPro NanoStar for DLS measurement (Wyatt, Santa Barbara, United States).

### Quantitative Real-Time PCR

RNA was extracted from exosomes or cells using a Quick-RNA MagBead kit (Zymo Research, Orange, CA) according to the manufacturer’s protocol, and the concentration of total RNA was measured using the Nanodrop micro-spectrophotometer. TaqMan MicroRNA Reverse Transcription kit (Thermo Fisher Scientific Carlsbad, CA, United States) was used to obtain single-stranded cDNA. qRT-PCR was then performed using TaqMan MGB probes (Applied Bioscience) for miR-26a-5p. Amplification was carried out using TaqMan Universal PCR Master Mix (Applied Biosystems) for 40 cycles. The levels of miR-26a-5p were measured using stem-loop qPCR. The data obtained after three independent experiments were analyzed using the relative quantitative (RQ) formula RQ = 2^−ΔΔCq^. U6 was used as the reference gene for normalization. Each sample was repeated three times. The primers used were as follows: miR-26a-5p-forward: 5ʹ-UCC AUA AAG UAG GAA ACA CUA CA-3ʹ, reverse: 5ʹ-CAG UAC UUU UGU GUA GUA CAA-3ʹ, and U6-forward: 5ʹ-CTC GCT TCG GCA GCA CAT ATA CT-3ʹ, reverse: 5ʹ-ACG CTT CAC GAA TTT GCG TGT C-3ʹ.

### Cellular Uptake of Exosomes

Carboxyfluorescein succinimidyl ester (CFSE) was used to stain the exosomes. Briefly, 2.5 μM CFSE was added into extracted 100 μg exosomes, supplemented with PBS to 50 μL, and incubated in a dark room at room temperature without stirring for 60 min. During incubation, the Exosome Spin Columns (Invitrogen) kit was prepared according to the instructions and the powder resin was hydrated at room temperature for 15–30 min. The incubated CFSE-labled exosomes were then added to the resin. Centrifuge the column in a 1.5 ml microcentrifuge tube at 750 × g for 3 min, and excess free dye could be absorbed by the resin. Fluorescence labeled exosomes were slowly added with 100 μL DMEM without phenol red, filtered in a 0.22 μm filter membrane, and then separated and stored at −80°C for later use. HepG2, Huh7 and 293T cells were seeded into 12-well plates at a concentration of 2 × 10^5^ cells/well. After being cultured overnight, the cells were treated with CFSE-labeled scFv-Exo^miR-26a^ (10 and 20 μg) for 6 h. The green fluorescence signals were observed and photographed under an inverted fluorescence microscope (Olympus Corp.). Then cells were digested and analyzed using the Cytoflex flow cytometer (Beckman Coulte) to measure the fluorescence intensity.

### MTT Assay

HepG2, Huh7, and 293T cells were seeded in 96-well plates with 3,000 cells per well and then treated with nc-Exo^miR-26a^ (unmodified MSC-derived exosomes carrying miR-26a), nc-Exo^miR-con^ (unmodified MSC-derived exosomes carrying control miRNA), scFv-Exo^miR-26a^ (anti-GPC3 scFv-modified exosomes carrying miR-26a) and scFv-Exo^miR-con^ (anti-GPC3 scFv-modified exosomes carrying control miRNA) for 0, 24, 48, 72, and 96 h. The MTT solution (5 mg/ml, 20 μL) was added, and the cells were incubated for an additional 4 h. Then, the medium was replaced with dimethyl sulfoxide (200 μL, DMSO, Sigma, St. Louis, Missouri, United States). The absorbance of the resulting solution was measured at a wavelength of 450 nm using a microplate reader.

### Colony-Forming Assay

A total of 10^3^ HepG2 and Huh7 cells were inoculated in six-well plates. After an overnight culture, the cells were treated with scFv-Exo^miR-26a^ and scFv-Exo^miR-con^. After 2 weeks of continuous culture, the cell supernatant in each well was discarded, and cells were rinsed twice with PBS. Four percent paraformaldehyde was added to fix cells for 15 min at 25°C, and the cells were then stained with 2 ml 0.5% crystal violet for 30 min at 25°C. After being dried, the plates were photographed, and the clones were counted.

### Scratch Wound Assay

Cell mobility was measured *via* a scratch wound-healing assay. HepG2 and Huh7 cells were inoculated in 12-well plates overnight. A 200 µl pipette tip was used to make scratches on the single cell layer. After gentle washes with PBS, the cells were then cultured in a cell incubator for another 48 h. Cells were photographed (0 and 48 h cell scratches) and the scratch area was measured using Image J software. Migration area ratio = (0 h scratch area − 48 h non migration area)/0 h scratch area.

### *In Vivo* Study

In order to determine the performance of scFv-Exo^miR-26a^ *in vivo*, 4–5 weeks old female B-NDG mice (NOD-*Prkdcscid Il2rgtm1*/Bcgen, Biocytogen Jiangsu Co., Ltd, Jiangsu, China) bearing HCC were established by subcutaneously injection of HepG2/luciferase cells (1 × 10^6^) in the right flank. Mice were divided into two groups randomly, and administered intratumorally for three times (on day 0, 3, 5) with scFv-Exo^miR-26a^ and scFv-Exo^miR-con^ at 100 μg/mouse, respectively. Afterwards, mice were imaged every 7 days by IVIS live imaging, and raised tumors were measured using a caliper according to the following formula: V = length × width^2^/2. The body weight was measured simultaneously as an indicator of systemic toxicity. At the end of the experiments, the mice were sacrificed, and tumors were resected, photographed and weighed, their organs (liver and kidney) were stained with hematoxylin-eosin (HE) to check for toxicity. The study was approved by the ethics committee of Xinjiang Medical University, and laboratory animal care as well as the user guide were followed. All animals were kept in accordance with Chinese animal health and welfare norms.

### Statistical Analysis

Data were analyzed and graphed using GraphPad Prism 9 (GraphPad Software, Inc., CA, United States). All data were presented as means ± SEMs. The two groups were statistically compared using the Welch’s unpaired *t*-test. Multiple groups were analyzed using one-way ANOVA. A *p*-value <0.05 was considered statistically significant.

## Results

### Schematic Illustration of the Exosome-Based Drug Delivery Systems

**FIGURE 1:**
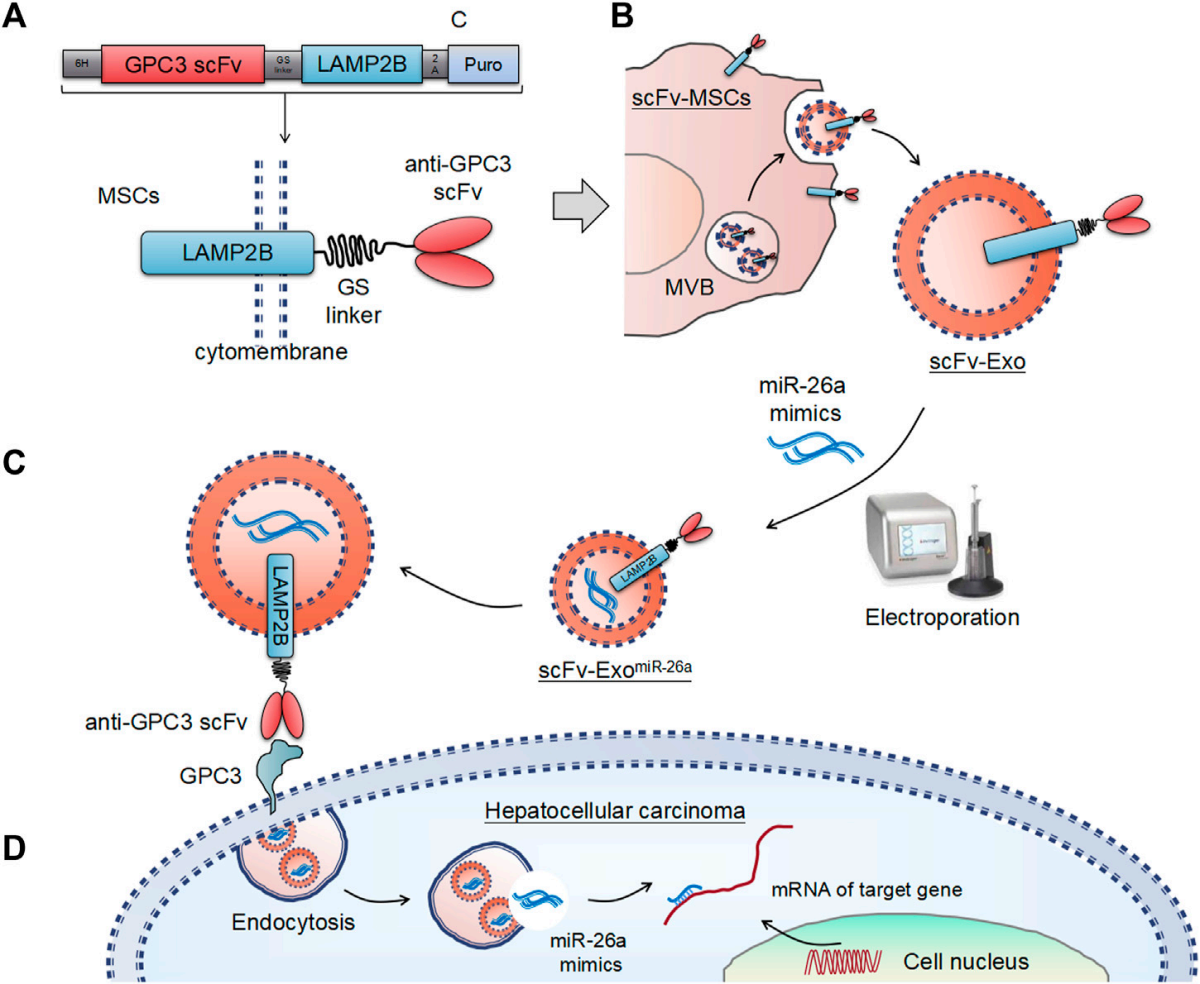
Schematic illustration of the exosome-based drug delivery systems. **(A)** Design of the anti-GPC3 scFv-Lamp2b fusion constructs. **(B)** Schematic representation of the exosomes-based nanosystem preparation. **(C)** Uptake of anti-GPC3 scFv-modified exosomes by tumor cells. **(D)** Schematic illustration of the antitumor mechanisms of miR-26a mimics.

Schematic illustration of the preparation of anti-GPC3 scFv-modified exosomes and anticancer drug delivery for the suppression of hepatocellular carcinoma were showed in Figure 1. The structural diagram of the anti-GPC3 single-chain antibody (scFv) and the lysosome-associated membrane protein 2b (LAMP2B) fusion gene was presented in Figure 1A. Fusion constructs were configured 5′→3′ as per the following: a signal peptide (SP), an anti-GPC3 single-chain antibody (scFv), a (Gly4-Ser)3 linker, the full-length Lamp2b lacking the endogenous SP sequences, followed by an in-frame self-cleaving peptide from porcine teschovirus-1 (P2A), a puromycin-resistant gene, and a stop codon. Fusion of a protein of interest to Lamp2b is very common for displaying the protein on the surface of exosomes. A Lamp2b-based fusion protein can be used to display anti-GPC3 scFv on the surface. Figure 1B showed a proposed model illustrating how the anti-GPC3 scFv-Lamp2b fusion protein participates in exosomes in mesenchymal stem cells (MSCs). Ectopic expression of the fusion protein occurs at the rough endoplasmic reticulum (ER) and becomes concentrated in lamp2b-enriched exosomes, which are that are stored in a multivesicular bodies (MVB) prior to release from the cell. The scFv-modified exosomes (scFv-Exos) were isolated from the supernatant *via* ultracentrifugation, and then transfected with miR-26a mimics using electroporation. The miR-26a-loaded exosomes (scFv-Exo^miR-26a^) were then uptake by hepatocellular carcinoma by binding to GPC3 on the tumor cell surface (Figure 1C). The exosomes were then incorporated into HCC cells by endocytosis, and the miR-26a mimics were then released and bind to the 3’ untranslated regions (UTRs) of its target mRNAs to suppress expression (Figure 1D).

### Identification of Exosomes Loaded With miR-26a Mimics

**FIGURE 2:**
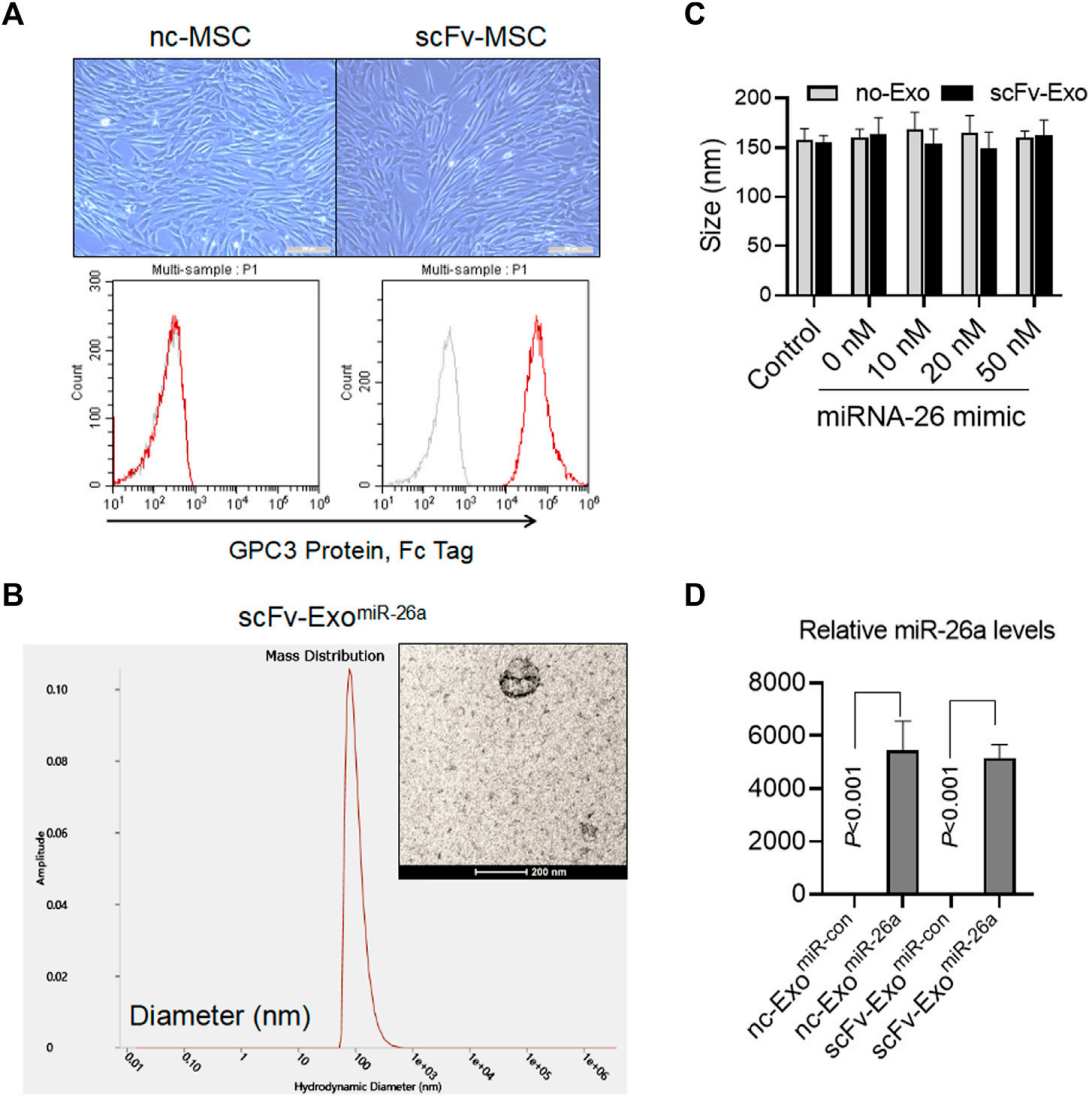
Identification of exosomes loaded with miR-26a mimics. **(A)** Morphological characteristics of the fusion gene-modified MSCs were observed, and the expression of anti-GPC3 scFv on the surface of MSCs was detected by flow cytometry. Scale bars = 50 μm. **(B)** The morphology of exosomes loaded with 20 nM miR-26a mimics (scFv-Exo^miR-26a^) as determined by electron microscopy. The particle size of exosomes was evaluated using dynamic light scattering. Scale bars = 200 nm. **(C)** Different amounts of miR-26a mimics (0–50 nM) were electrotransferred into exosomes, and the particle size of exosomes was evaluated using dynamic light scattering. **(D)** Detection of miR-26 levels in engineered exosomes using quantitative real-time PCR (qRT-PCR).

The fusion gene of anti-GPC3 scFv-Lamp2b was packaged with lentivirus and used to infect the mesenchymal stem cells (MSCs). After 14 days of selection using puromycin, the anti-GPC3 scFv-modified MSCs (scFv-MSCs) were obtained. Compared with the unmodified negative control MSCs (nc-MSCs), scFv-MSCs did not exhibit any changes in morphology and had the typical MSC fusiform shape (Figure 2A). In addition, flow cytometry analysis indicated that anti-GPC3 scFv was highly expressed in scFv-MSCs by detecting their binding with GPC3 protein (Figure 2A). Then, miR-26a mimics were transfected into the exosomes derived from scFv-MSCs using electroporation, and the resulting morphology is presented in the Figure 2B. The particle size of the scFv-Exo^miR-26a^ exosome was evaluated by DLS, with a size of approximately 160 nm. Moreover, electroporation of different amounts of miR-26a mimics (0–50 nM) did not affect the particle size of exosomes (Figure 2C). The levels of the miR-26a loaded in the exosomes were further confirmed by qRT-PCR, and the results indicated that miR-26a levels were markedly higher in miR-26a-transfected nc-Exo and scFv-Exo than those in miR-con-transfected nc-Exo and scFv-Exo, respectively (all *p* < 0.001, Figure 2D).

### Exosomes-Based Nanosystem Effectively Delivered miR-26a Into Hepatocellular Carcinoma Cells

**FIGURE 3:**
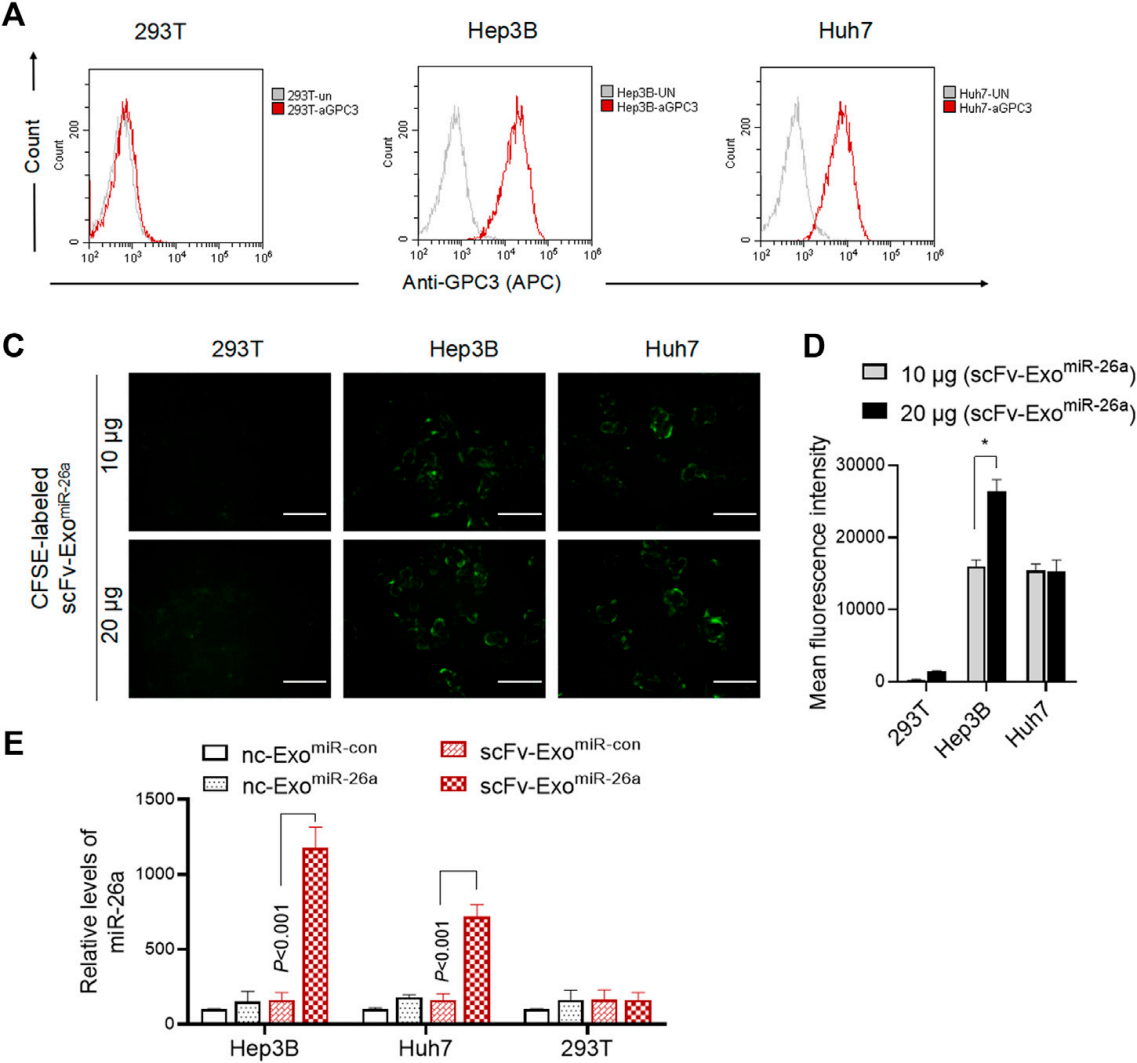
Exosomes-based nanosystem effectively delivered miR-26a into HCC cells. **(A)** Flow cytometric detection of the expression of GPC3 antigen protein on the surface of 293T, HepG2 and Huh7 cells. **(B)** And the percentage of HCC cells expressing GPC3 was showed. ***p < 0.001 vs 293T. **(C)** CFSE-labeled scFv-Exo^miR-26a^ (10 and 20 μg) exosomes were added into 293T, HepG2 and Huh7 cells, and the green fluorescence signals were observed and photographed after 6 h. Scale bars = 50 μm. **(D)** The fluorescence intensity of CFSE was measured by flow cytometry. ***p < 0.001 vs 293T. **(E)** Detection of the abundance of miR-26a mimics in cells using quantitative real-time PCR (qRT-PCR).

We next examined whether the drug-loaded exosomes could improve the miR-26a expression in HCC cells. Firstly, we evaluated the expression of GPC3 on the surface of human HCC lines. Indeed, HepG2 and Huh7 cells, rather than 293T, exhibited higher levels of GPC3 expression (Figures 3A,B). It was revealed that CFSE-labeled scFv-Exo^miR-26a^ was only able to be uptake by both HepG2 and Huh7 tumor cells, and effectively displayed green fluorescence (CFSE) (Figures 3C,D). After co-culture with exosomes for 24 h, the levels of miR-26a expression were higher in the scFv-Exo^miR-26a^-treated HepG2 and Huh7 cells than in the GPC3 negative 293T cells (Figure 3E). Notably, the exosomes derived from the unmodified negative control MSC (nc-Exo) was not effective in delivering miRNAs to tumor cells.

### Exposure to miRNA Drug-Loaded Exosomes Inhibited Hepatocellular Carcinoma Cells Proliferation

**FIGURE 4:**
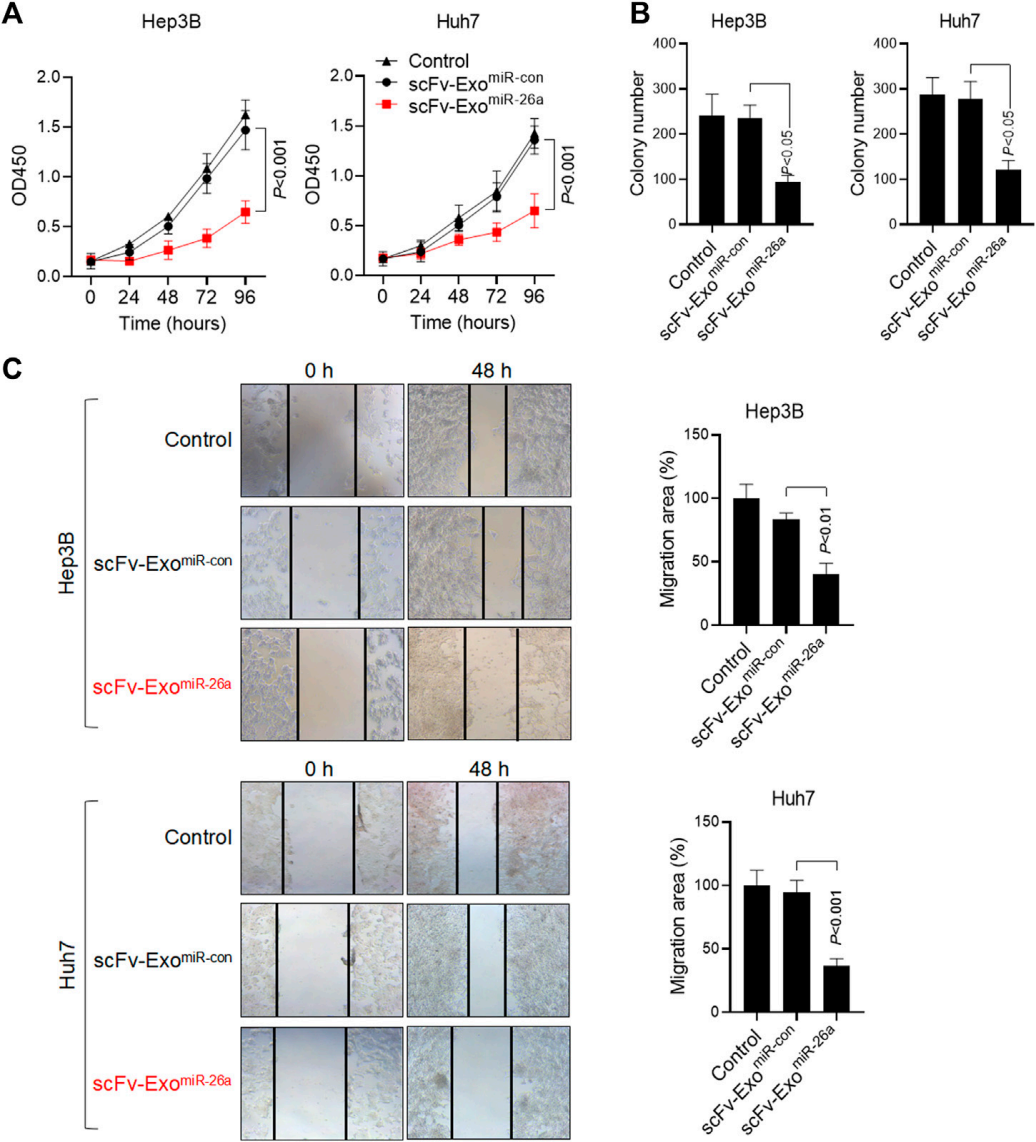
Exposure to miRNA drug-loaded exosomes inhibited HCC proliferation. **(A)** MTT detection of cell proliferation in 293T, HepG2, and Huh7 cells after the addition of 20 μg miRNA drug-loaded exosomes. **(B)** The number of cell clones was counted after the addition of drug-loaded exosomes. **(C)** The migration of HepG2 and Huh7 cells in different treatment groups was detected by scratch assay (100×). Relative migration area was quantified (bar charts).

To determine the effects of modified exosomes on the proliferation of HepG2 and Huh7 cells *in vitro,* the cell lines were treated with scFv-Exo^miR-26a^ and scFv-Exo^miR-con^. The cell viability of HepG2 and Huh7 cells was not altered after scFv-Exo^miR-con^ treatment, whereas scFv-Exo^miR-26a^ obviously inhibited cell proliferation of both HepG2 and Huh7 cells (Figure 4A). Clone formation also revealed a decrease in the number of cell clones among scFv-Exo^miR-26a^-treated HepG2 and Huh7 cells, as compared to scFv-Exo^miR-con^-treated cells (Figure 4B), thus suggesting that scFv-Exo^miR-26a^ could effectively inhibit tumor cell proliferation in HCC. Cell mobility was measured *via* a scratch wound-healing assay, and the results demonstrates that exposure to scFv-Exo^miR-26a^ inhibited the cell migration in both HepG2 and Huh7 cells (Figure 4C).

### Exosomes-Based Nanosystem Displayed Favorable Anti-Tumor Effect *in Vivo* With No Obvious Side Effects

**FIGURE 5:**
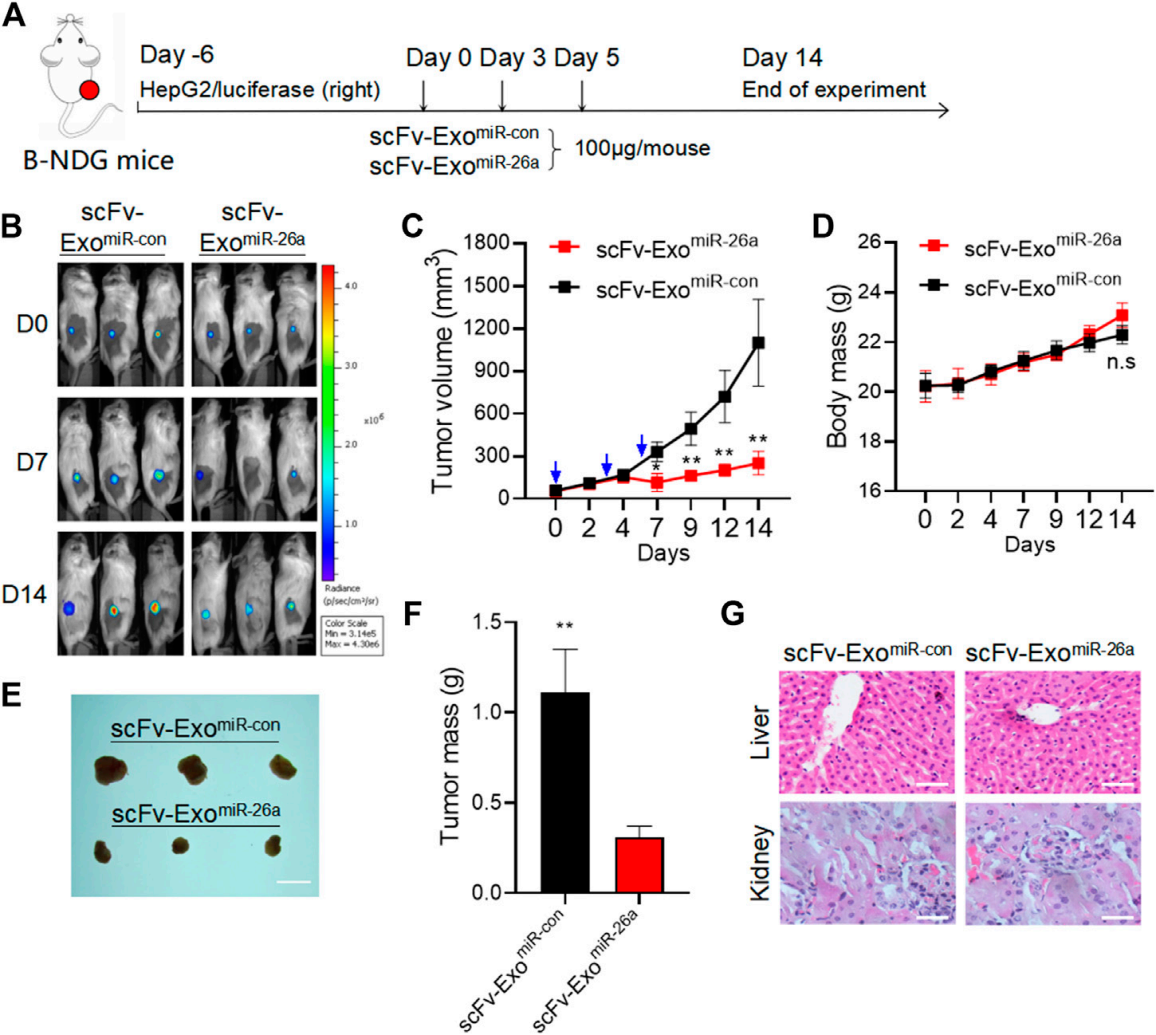
Exosomes-based nanosystem displayed favorable anti-tumor effect *in vivo* with no obvious side effects. **(A)** Schematic representation of the experimental arrangement for animal experiments. **(B)** *In vivo* live imaging of mouse with HCC tumor formed by HepG2/luciferase cells. **(C)** Tumor volume was measured over time. *p < 0.05, **p < 0.01. **(D)** These mice were fed for 14 days for weight analysis. **(E)** Tumors collected from mice were exhibited. Scale bars = 1 cm. **(F)** Average tumour weight in each group was calculated. **p < 0.01. **(G)** Mice were sacrificed after *in vivo* imaging, and HE staining of liver and kidney tissues was then performed. Scale bars = 50 μm.

To evaluate the antitumor effects of exosomes-based nanosystem *in vivo*, B-NDG mice bearing HepG2/luciferase cells were subjected to three treatments with scFv-Exo^miR-26a^ and scFv-Exo^miR-con^ at 100 μg/mouse within 7 days, respectively (Figure 5A). The results of live-imaging showed lower fluorescent signals in mice receiving scFv-Exo^miR-26a^ than that in scFv-Exo^miR-con^ treated mice (Figure 5B). The tumor volume was smaller in the mice treated with miR-26a-loaded exosomes compared to control group (Figure 5C). Meanwhile, all mice had a normal diet and their body weights increased in the whole process of experiment, and the body weights of the two groups showed no significant change (Figure 5D). At the end of the experiments, the tumor size (Figure 5E) and tumor weight (Figure 5F) were significantly decreased, but the liver and kidney (Figure 5G) had no morphological differences, when compared to the control.

## Discussion

Although miRNAs have been proved to be potential antitumor drug candidates, the lack of a specific delivery system has impeded their clinical application. In this study, we found that antibody-modified exosomes, as a drug delivery nanosystem, could represent a potential carrier to efficiently deliver antitumor miRNAs to HCC.

Exosomes are a nano-sized follicles originating from cell endocytosis and are released by different cell types (Koppers-Lalic et al., 2013). Exosomes essentially act as a communication medium between cells by delivering protein and RNA (Vlassov et al., 2012). Therefore, the application of exosomes as a biological delivery medium is very promising. Accumulating research has indicate that MSCs are highly suitable for the mass production of exosomes, and artificially modified exosomes can be obtained by harvesting supernatants from genetically modified MSC cells (Phan et al., 2018). When antibodies are fused with LAMP2B, an exosome-associated transmembrane protein, they are also highly efficiently expressed on the surface of the exosomes (Limoni et al., 2019). In the present study, genetic engineering and protein technology were used to obtain anti-GPC3 scFv-modified exosomes for GPC3-specific targeting and drug delivery.

miRNAs exert a strong influence on cell behavior through the regulation of extensive gene expression networks (Rottiers and Näär, 2012). Therefore, the therapeutic regulation of one miRNA can affect multiple pathways and, at the same time, can achieve clinical benefits. To date, most *in vivo* translation studies investigating miRNAs have been aimed at preparations using antisense reagents (such as locked nucleic acid oligomers and other qualified oligonucleotides), which inhibit the function of endogenous miRNAs (Van Rooij, 2011). However, most tumors are characterized by an overall decrease in miRNA expression compared to their normal counterpart tissues, and experimental interference with miRNA expression has been demonstrated to promote cell transformation and tumorigenesis (Xu et al., 2006; Paquet et al., 2016). Furthermore, carcinogenic lesions can result in widespread miRNA inhibition (Berti et al., 2019). Therefore, targeted miRNA delivery will enable therapeutic recovery of physiological regulatory programs lost in cancer and other disease conditions.

MiR-26a is a potentially excellent miRNA proposed as a treatment for hepatoma cancer (Yang et al., 2013). MiR-26 family members have multiple antitumor properties in various tumor contexts (Yang et al., 2014). In liver cancer, miR-26a was reported to induce cell-cycle arrest associated with direct targeting of cyclins D2 and E2. However, if future studies determine that the role of miR-26 delivery is selective for the treatment of Myc disorders, then the efficient delivery of this miRNA remains an important objective to achieve, as the therapeutic delivery of this miRNA will be effective for a large number of human cancer subtypes (Paquet et al., 2016). Since physiological gene expression networks have evolved to adapt to endogenous miRNA regulation, the risk of off-target gene silencing may be less than the risks associated with artificial RNAi triggers (Xu et al., 2006). In this study, we designed a drug delivery system that used anti-GPC3 scFV-modified exosomes to load miR-26a and inhibited the proliferation and migration of GPC3-positive HCC cells *in vitro*. To further evaluate the efficacy of exosomes-based nanosystem in HCC, we conducted an antitumor efficacy study in a HepG2 cell mouse xenograft model. We found that nanosystem mediated miR-26a delivery effectively enhanced the curative effects in HCC tumor, which coincided with the results from those results *in vitro*. Also, a future comparison between intravenous and intratumoral viral administration may be warranted.

## Conclusion

Our study indicated that miR-26a delivery *via* scFv-modified exosome carriers could inhibit the growth of HCC cells and thus represents a new treatment strategy for hepatoma. Technological improvements aimed at enhancing the preparation of exosomes and reducing potential immunogenicity should be explored to further develop this effective method of drug delivery.

## Data Availability

The original contributions presented in the study are included in the article/Supplementary Material, further inquiries can be directed to the corresponding authors.
